# Recycling Coal Fly Ash for Super-Thermal-Insulating Aerogel Fiber Preparation with Simultaneous Al_2_O_3_ Extraction

**DOI:** 10.3390/molecules28247978

**Published:** 2023-12-06

**Authors:** Jie Gu, Lipeng Liu, Rongrong Zhu, Qiqi Song, Hanqing Yu, Pengjie Jiang, Changqing Miao, Yuxiang Du, Rui Fu, Yaxiong Wang, Yan Hao, Huazheng Sai

**Affiliations:** 1 School of Chemistry and Chemical Engineering, Inner Mongolia University of Science & Technology, Baotou 014010, China; gujie22@mails.ucas.ac.cn (J.G.); lipengliu1998@163.com (L.L.); rzr1208@163.com (R.Z.); songqiqiaa@163.com (Q.S.); q1252051550@163.com (H.Y.); jpj1692787089@163.com (P.J.); qingmc@163.com (C.M.); duyuxiang5520@163.com (Y.D.); wangyaxiong2021@126.com (Y.W.); haoyannk@163.com (Y.H.); 2 Inner Mongolia Key Laboratory of Coal Chemical Engineering & Comprehensive Utilization, Inner Mongolia University of Science & Technology, Baotou 014010, China; 3 Aerogel Functional Nanomaterials Laboratory, Inner Mongolia University of Science & Technology, Baotou 014010, China

**Keywords:** silica aerogel fiber, coal fly ash, bacterial cellulose, high strength, thermal insulation, Al_2_O_3_

## Abstract

A large quantity of coal fly ash is generated worldwide from thermal power plants, causing a serious environmental threat owing to disposal and storage problems. In this work, for the first time, coal fly ash is converted into advanced and novel aerogel fibers and high-purity α-Al_2_O_3_. Silica–bacterial cellulose composite aerogel fibers (CAFs) were synthesized using an in situ sol-gel process under ambient pressure drying. Due to the unique “nanoscale interpenetrating network” (IPN) structure, the CAFs showed wonderful mechanical properties with an optimum tensile strength of 5.0 MPa at an ultimate elongation of 5.8%. Furthermore, CAFs with a high porosity (91.8%) and high specific surface area (588.75 m^2^/g) can inherit advanced features, including excellent thermal insulation, stability over a wide temperature range, and hydrophobicity (contact angle of approximately 144°). Additionally, Al_2_O_3_ was simultaneously extracted from the coal fly ash to ensure that the coal fly ash was fully exploited. Overall, low-cost woven CAFs fabrics are suitable for wearable applications and offer a great approach to comprehensively use coal fly ash to address environmental threats.

## 1. Introduction

Coal fly ash, a type of solid waste released by factories and thermal power plants, mainly contains a SiO_2_–Al_2_O_3_ mixture [1,2,3]. Over the past few decades, the emissions from and disposal of coal fly ash have caused significant economic and environmental problems [4,5]. Currently, the comprehensive utilization of coal fly ash mainly focuses on concrete, bricks, adsorbents, insulation panels, and agricultural fertilizers, which lack high-value applications [6,7]. Although the extraction of Al_2_O_3_ is an effective means to realize the high-value utilization of coal fly ash [8], such methods often fail to realize the effective utilization of SiO_2_, which is one of its main components; this not only fails to completely realize the true harmless utilization of coal fly ash but also greatly reduces its economic value. Therefore, it is necessary to develop a simple but efficient approach to extract Al_2_O_3_ from coal fly ash and simultaneously achieve the high-value utilization of SiO_2_.

SiO_2_ aerogels have attracted much attention in academia and industry because of their excellent properties, such as high porosity (80–99.8%), ultralow density (0.003–0.1 g/cm^3^), low thermal conductivities (e.g., 12–20 mW/m·K), high specific surface area (500–1200 m^2^/g), and good chemical stability (e.g., fire and corrosion resistance) [9,10,11,12]. Especially, SiO_2_ aerogels satisfy the requirements of super thermal insulation and are lightweight, representing state-of-the-art thermal insulation material [13,14,15,16]. Nonetheless, their product forms, which usually appear in blankets, monoliths, and powders, are relatively simple to prepare with traditional chemical precursors (e.g., ethyl orthosilicate and sodium silicate) [17] or solid wastes (fly ash, coal gangue, and kaolin) [2,4,18,19,20], which results in their highly fragile nature. Consequently, inorganic aerogels have significant limitations regarding their use in many application scenarios. Effective thermal protection of clothing is crucial for personal safety, especially for people who are consistently exposed to excessively low or high temperature conditions, such as polar researchers, divers in the deep sea, firefighters in fire rescue, and steelworkers [21,22,23]. However, natural fibers (e.g., cotton, fibrilia, wool, silk, or down) or synthetic fibers (e.g., polyester, nylon, or polyurethane) suffer from the disadvantages of limited porosity and relatively high thermal conductivity (usually larger than 0.06 W/m·K) [24,25,26,27]. Ordinary textiles made from these fibers do not meet the thermal protection requirements for complex applications in extreme environments [28,29,30]. Hence, thicker and heavier textiles are required to provide effective thermal comfort, which is inconsistent with the current demand for lightweight, simple, and convenient clothing. Therefore, it is essential that SiO_2_ aerogels, which are light and heat-insulating materials, are used in the textile industry.

Considerable research has been conducted to use SiO_2_ aerogel processing in textiles. The most direct approach involves the formation of an aerogel on a textile. Oh et al. used the direct gelation of SiO_2_ on PET to obtain a flexible PET/aerogel blanket [31]. In addition, processed aerogels (e.g., SiO_2_ aerogel paste) can be applied to textiles, often via fixation by melt bonding or polymer binder [32]. A related approach involves combining polymers and aerogels in a membrane and incorporating the membrane into a fabric assembly [33,34]. In a novel approach for combining aerogels with textiles, Xiong et al. used a laser to create voids in both melt-bonded nonwoven polyester fabric and polyurethane open-cell foam, and the voids were filled with hydrophobic silica aerogel to obtain aerogel-encapsulated textiles [35]. Nevertheless, rather than directly applying an aerogel or aerogel precursors to a textile, SiO_2_ aerogels are best introduced in fiber form. This is attributed to two reasons: (1) the fiber form facilitates the preparation of textiles of various shapes, and (2) SiO_2_ fibers can be combined with other fibers to create more comfortable and thermally insulating fabrics. Recently, several studies have reported the use of SiO_2_ aerogel fibers, such as hollow SiO_2_ aerogel fibers [36] and transparent SiO_2_ aerogel fibers [16]. However, their tensile strength generally does not exceed 0.5 MPa, making it difficult to meet the requirement applied in textiles. Consequently, further research is required to improve the mechanical properties of inorganic aerogels fibers to significantly enhance the likelihood of weaving. Considering the inherent high degree of flexibility of organic materials, the use of polymers could be an effective strategy to improve the mechanical properties of SiO_2_ aerogel fibers.

Cellulose is the most abundant biopolymer that meets the demand for environmentally friendly degradable and renewable products [10,37,38,39], and it is mainly used to toughen aerogels into blankets and monolith shapes [40,41,42]. However, reports on the toughening of cellulose aerogels into fiber forms are still rare. In our previous study, we successfully prepared flexible aerogel fibers based on an interpenetrating network (IPN) of bacterial cellulose (BC) and silica aerogels, which confirmed that the incorporation of three-dimensional (3D) aerogels into cellulose or filaments is attractive [43]. Silica–BC composite aerogels with an IPN structure of soft organic and rigid inorganic components on a molecular scale have been synthesized to overcome the lack of flexibility and robustness of inorganic materials. As a result, it is of great significance to further explore new synthetic strategies for preparing high-performance thermal insulation silica–cellulose composite aerogel fibers (CAFs) based on nanoscale interpenetrating networks.

In this work, a method for extracting Al_2_O_3_ from coal fly ash and transforming the SiO_2_ contained therein into aerogel fibers is proposed. To obtain aerogel fibers with excellent mechanical properties, BC was used as a reinforcing matrix to form a composite with a SiO_2_ gel skeleton to form an IPN structure at the nanoscale. The unique nanoscale IPN structure endows the silica–BC CAFs with excellent mechanical properties, such as an optimum tensile strength of 5.0 MPa at an ultimate elongation of 5.8%. In addition, these CAFs surfaces are hydrophobic and exhibit unique aerogels with ultra-high porosity (91.8%), high specific surface area (588.75 m^2^/g), and remarkable thermal insulation properties. This work not only offers inspiration for the preparation of a family of fibrous aerogel materials for use in thermal insulation but also paves the way for an effective way to comprehensively utilize coal fly ash.

## 2. Results and Discussion

### 2.1. Morphologies and Structure of CAFs

**Figure 1:**
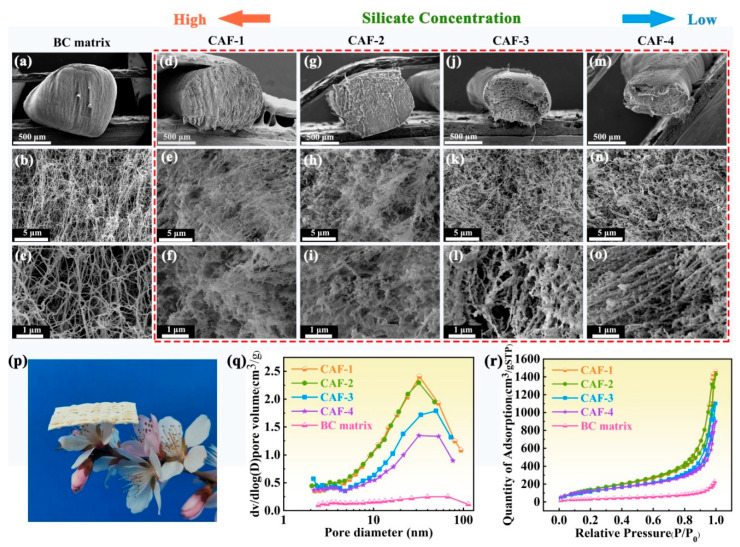
(**a**–**c**) SEM images of the BC matrix, magnified at 50×, 5000×, and 20,000×, respectively. (**d**–**f**), (**g**–**i**), (**j**–**l**), and (**m**–**o**) are SEM images of CAF-1, CAF-2, CAF-3, and CAF-4, respectively, magnified at 100×, 5000×, and 20,000×. (**p**) Photograph of a woven fabric composed of CAF-1 perched on peach blossoms. (**q**) Nitrogen adsorption–desorption isotherms and (**r**) pore size distribution of the BC matrix and CAFs.

As observed in the SEM images (Figure 1d–o), the presence of SiO_2_ gel frameworks within all the CAFs was evident compared to the BC matrix (Figure 1a–c). CAF-1 (Figure 1d) has a diameter (approximately 0.8 mm) similar to that of the BC matrix (Figure 1a), suggesting minimal shrinkage during the ambient drying process. However, CAF-2 displayed slight shrinkage owing to the lower SiO_2_ sol (SS) concentration (Figure 1g), whereas CAF-3 and CAF-4 experienced severe shrinkage, even though their cross-sections were no longer circular (Figure 1j,m). Furthermore, the silica gel skeletons of CAF-1 and CAF-2 prepared with high concentrations of SS were more compact and formed a uniformly distributed dual network structure with BC fibers (Figure 1f,i), indicating that the IPN structure of a rigid SiO_2_ gel skeleton and a flexible BC nanofiber network was formed. This is because the cavity of the matrix is large enough to allow for the formation of a silica gel skeleton network almost without interference as the distance between the BC nanofibers is at the microscale (Figure 1c) and does not only lead to the adherence of silica nanoparticles to the matrix network to form an isotropic network [44]. In addition, the diameter of the BC gel skeleton closely resembles that of the silica gel skeleton, which facilitated the development of the IPN structure [45]. The distinctive microstructural attributes resulted in satisfactory lightweight characteristics, which allowed the woven fabric composed of CAF-1 to easily perch on the slender pistil of the peach blossoms (Figure 1p). In contrast, CAF-3 and CAF-4 showed SiO_2_ nanoparticles that were primarily stacked or loosely adhered to the BC fibers without forming an effective 3D network structure (Figure 1l,o). This phenomenon can be attributed to the silanol groups (Si–OH) on the surfaces of the silica nanoparticles and the hydroxyl groups (–OH) on the surfaces of the cellulose fibers (Figure S5), which promoted the adhesion of SiO_2_ nanoparticles to the cellulose fiber surfaces. Consequently, the significant consumption of silica nanoparticles on the fiber surface makes it challenging to form a substantial and well-developed gel skeleton within the BC matrix when the concentration of silica precursors is relatively low. Moreover, the rigid gel skeleton underwent shrinkage followed by rebounding during the ambient drying process [45]. As a result, the CAF-3 and CAF-4 experienced more significant shrinkage because their inadequate mechanical strength resulted in it being difficult for the few gel skeletons to withstand the shrinkage caused by the capillary force and to fail to spring back to their original shape.

The nitrogen adsorption–desorption isotherms of the CAFs demonstrated type Ⅳ behavior (Figure 1r) according to the IUPAC classification, which is a typical profile for mesoporous materials with a characteristic hysteresis loop [46]. Conversely, the nitrogen adsorption–desorption isotherm and pore size distribution analysis revealed the scarcity of a mesoporous structure within the BC matrix (Figure 1q,r). This suggested that the incorporation of the silica gel skeleton imparts a mesoporous structure to the CAFs. Remarkably, CAF-1 and CAF-2 exhibit more pronounced hysteresis loops than CAF-3 and CAF-4, and the pore size distributions (Figure 1q) illustrate that the mesoporous structure of CAF-4 is the least prominent. In addition, a Brunauer–Emmett–Teller (BET) analysis revealed a decrease in the specific surface area from 588.75 to 234.81 m^2^/g (Table 1) with decreasing SS concentration. This could be rationalized as follows: the nanoparticles were primarily stacked or loosely adhered to the matrix without forming an effective 3D network structure when the SS content was low (Figure 1l,o). An increase in the mass proportion of cellulose corresponds to a decrease in the specific surface area. These observations underscore the substantial influence of the silica precursor concentration on the pore structure of CAFs, highlighting the significance of precise control over the precursor level to attain the optimal microstructure.

### 2.2. Hydrophobization of the Surfaces of CAFs

**Figure 2:**
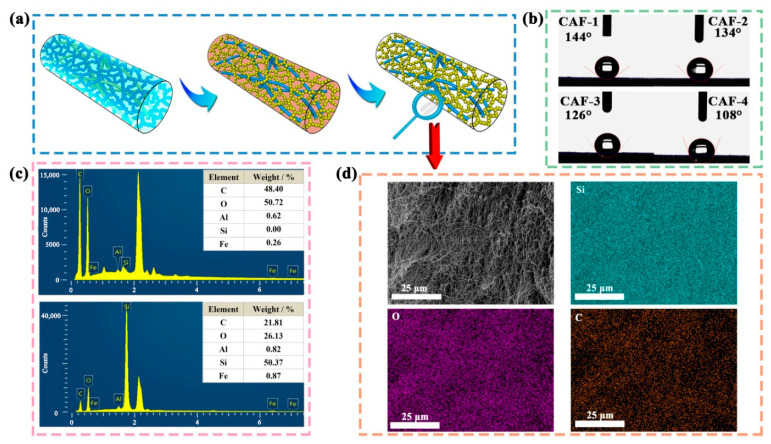
(**a**) Illustration of the hydrophobic modification process of CAFs. (**b**) Wettability of CAF1–4. (**c**) EDS spectra of CAF-1 and BC matrix with weight concentration for C, O, Si, Al, and Fe. (**d**) EDS elemental (C, O, Si, Al, and Fe) mapping images of CAF-1.

To ensure effective spring-back of the gel skeleton during ambient drying and to protect the nanopore structure of the CAFs from water vapor damage, broadening its potential applications, a hydrophobic modification was performed on the wet gel fibers. This involved substituting the hydroxyl groups (–C–OH and –Si–OH) on the surface of the BC nanofibers and silica gel skeleton with inert and hydrophobic methyl groups [47,48]. The hydrophobic modification process (Figure 2a) effectively impeded the further formation of new –Si–O–Si bonds on the gel skeleton surface, ensuring that the gel skeleton springs back adequately [45,49]. In addition, the inert methyl groups prevented the further formation of hydrogen bonds and/or covalent bonds [50,51] between the BC matrix and SiO_2_ gel skeleton during the ambient drying process, which restricted thermal transport across their interface and ensured the superior thermal insulation performance of the CAFs.

As illustrated in Figure 2b, the contact angles of the CAFs were all greater than 100°, indicating that the gel skeletons of the CAFs demonstrated considerable hydrophobicity. Moreover, a notable decrease in the contact angle of the samples was observed as the amount of the silica precursor in the samples decreased. This phenomenon can be attributed to two factors. First, the outer surfaces of the CAFs without hydrophobic modification possessed both silica and carbon hydroxyl groups, which exhibited higher reactivity and interaction with TMCS during the hydrophobic modification process, contributing to a greater incorporation of hydrophobic alkyl groups on the outer surface when the silica content was high. Secondly, the shrinkage and inadequate presence of the gel skeleton resulted in the diminished surface roughness of the CAFs compared to the adequate nanoporous gel network. Therefore, the decreases in the hydrophobic groups and surface roughness [52] collectively contributed to a decrease in the hydrophobicity of the CAFs.

The EDS spectrum of CAF-1 (Figure 2c) showed that its silicon content was as high as 50%, while the aluminum and iron contents were less than 1%, indicating that the relevant elements could be effectively separated. It is noteworthy that the BC matrix itself did not contain any Si elements. These results further suggested the successful composite formation of silica elements with the BC matrix. The element mapping distribution (Figure 2d) revealed a relatively uniform dispersion of Si throughout the CAFs. This further indicated that the SiO_2_ aerogel was effectively and uniformly incorporated within the CAFs using the synthesis method employed. 

### 2.3. Mechanical Properties

**Figure 3:**
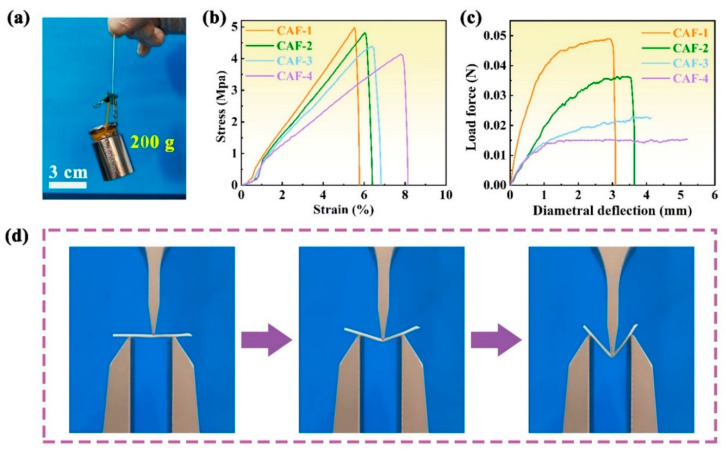
Mechanical performance of CAFs. (**a**) Single CAF-2 could bear a 200 g weight without breaking. (**b**) Stress–strain curves of tensile tests of the CAFs. (**c**) Three-point bending tests of the CAFs (fixture span: 20 mm). (**d**) Photographs of a three-point bending test for CAF-3.

The mechanical performance of CAFs with a nanoscale interpenetrating network structure was evaluated. Figure 3a shows that a single CAF could hold up to a 200 g weight without breaking, demonstrating its robust mechanical properties. The mechanical performance of CAFs prepared with different concentrations of the silica precursor is illustrated in Figure 3, demonstrating that all samples displayed excellent tensile strength. The CAFs exhibiting a tensile strength range of 4.2–5.0 MPa, significantly surpassing that of native silica aerogel fibers such as SiO_2_ aerogel fibers, which typically does not exceed 0.5 MPa [16,36]. The stress–strain curves of the CAFs show that the elongation at break of the samples decreases from 8.1% to 5.8% as the silica precursor concentration increases from 26 to 57 wt% (Figure 3b). This could be because when denser gel skeletons were formed between the BC nanofibers, the free movement of the nanofibers was restricted, resulting in the free deformation space of the nanofiber network being compressed. 

Three-point bending tests revealed similar results. CAF-1, which has the highest silica precursor content, exhibited less deformation than CAF-2, which has relatively low silica precursor content. Moreover, CAF-3 and CAF-4, with even lower silica precursor contents, exhibited no apparent fractures within a wide deformation range (Figure 3c,d and Video S1). This behavior can be attributed to the hypothesis that higher concentrations of the silica precursor led to the formation of more sufficient gel skeletons between the BC nanofibers, thereby restricting the free movement of the nanofibers and compressing the available deformation space. Furthermore, the stress required for fracture in the three-point bending test decreased with decreasing silica precursor content in CAF-1 and CAF-2. These tests further confirm that lower concentrations of the silica precursor result in lower brittleness, but also lead to decreased rigidity and resistance to external impacts in CAFs. Consequently, the precise control of the silica precursor concentration is crucial for the fabrication of aerogel fibers with exceptional mechanical properties.

### 2.4. Thermal Insulation Performance of CAFs

**Figure 4:**
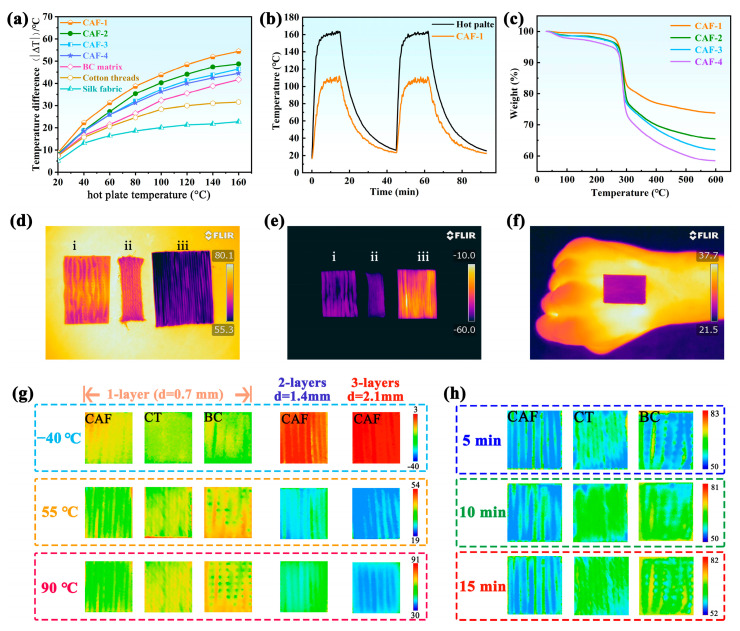
(**a**) Temperature difference between the fiber surface and hot plate versus temperature of the hot plate for the single-layer mats made of CAFs, BC matrix, CT, and silk fabric. (**b**) Temperature–time curves of CAF-1 and the hot plate. (**c**) TGA traces of CAFs. Infrared images of one-layer mat of (i) BC matrix, (ii) CT, and (iii) CAF-1 at (**d**) high (80 °C) and (**e**) low (–60 °C) temperatures. (**f**) Infrared images of the CAF-1 mat on a hand in the room-temperature the heat-shielding performance test. (**g**) Infrared images of mats from different sample fibers on different temperature plates for 10 min. From left to right: one-layer CAF-1, CT, and BC mats, followed by two- and three-layer mats from CAF-1. (**h**) Infrared thermal images of different mats variation with heating time at 80 °C heated plate.

Thermal insulation plays a significant role in maintaining normal physiological activities of the human body in excessively low- or high-temperature environments. Considering the potential application of aerogel fibers in the field of thermal insulation, the thermal insulation performance of CAFs obtained from different concentrations of SS was measured under both hot and cold conditions. For a comparative analysis, the BC matrix fiber, cotton threads (CTs), and silk fabric with diameters or thicknesses similar to those of the CAFs were subjected to identical testing conditions. As shown in Figure 4a, the temperature of a single-layer CAF-1 mat was measured to be 105 °C, whereas the surface temperatures of a single-layer BC matrix fiber mat, CT mat, and silk fabric mat were 117, 128, and 137 °C, respectively, at a heating temperature (Th) of 160 °C. The thermal insulation of the investigated materials improves with increasing temperature difference (|∆*T*|) [53]. Consequently, this result indicates that the CAFs exhibit superior thermal insulation properties compared with pure BC matrix fibers, CTs, and silk fabric. Furthermore, the thermal insulation performance of the CAFs improved gradually with an increase in the concentration of SS, which could be ascribed to the formation of effective 3D network gel skeletons rather than the simple adherence of silica nanoparticles to the matrix network.

To further investigate the stability of the insulation performance of the CAFs, an evaluation was conducted on the dynamic temperature changes occurring on the surfaces of the hot plate and CAF-1 during a heating-cooling cycle, as depicted in Figure 4b. The surface temperature of CAF-1 ranged from 18 to 110 °C, while that of the hot plate ranged from 18 to 165 °C. Once *T*_h_ stabilized at 165 °C, the |∆*T*| between the hot plate and CAF-1 was approximately 55 °C. Furthermore, when CAF-1 was reheated following the heating-cooling process, there was no discernible change observed in the |∆*T*|. These results provide clear evidence that the thermal insulation performance of CAF-1 remained stable.

The TGA traces of the CAFs are shown in Figure 4c. The thermal degradation of CAF 1–4 took place in three steps with similar weight losses: 30–250 °C, 250–300 °C, and 300–600 °C. In the first stage (30–250 °C), the degradation resulted from the evaporation-based loss of water vapor from the pores. Evidently, the hydrophobic-modified CAFs showed a significant reduction in the number of water molecules attached to the surface of the gel skeleton; thus, there was no obvious weight loss (exceeding 5 wt %). The main degradation of CAFs occurred in the second stage from 250 to 300 °C, originating from the combustion of the BC matrix skeleton in the CAFs. During the third stage (300–600 °C), a noticeable reduction in weight was also observed. This can be attributed to the substantial concentration of –Si–O–CH_3_ functional groups on the surface of the CAFs, which is a consequence of their hydrophobic modification. Upon reaching temperatures surpassing 400 °C, these functional groups undergo degradation [54]. It is worth noting that the residue of CAFs at 600 °C gradually decreased (CAF1–4) with decreasing concentrations of SS. Consequently, the presence of silica enhances the thermal stability of the polymer matrix. This enables the utilization of CAFs at elevated temperatures beyond the capabilities of conventional polymer fibers, which is attributed to the stabilizing influence of silica on the BC matrix.

To intuitively evaluate the thermal insulation properties of the CAFs, an infrared camera (FLIR) was used to monitor the surface temperature of a one-layer BC matrix, CT, and CAF-1 mat after being placed on surfaces at different temperatures. A sequence of infrared images was captured for 15 min. Figure 4d,e illustrate that the CAF mat is more effective at thermal insulation than the commercial CT and BC mats at relatively low (−60 °C) and high (80 °C) temperatures, despite having almost the same thickness (∼0.70 mm). In addition, thermal conductivity tests were conducted on the fabric, revealing that the woven fabric made from CAF-1 displayed a thermal conductivity of 0.0298 W/(m·K). Comparative analyses were also carried out with other fabric materials: a woven fabric consisting of a BC matrix displayed a thermal conductivity of 0.0506 W/(m·K), whereas a woven fabric made of CT exhibited a thermal conductivity of 0.0513 W/(m·K). This further test distinctly demonstrated that the aerogel-based fabric exhibited significantly lower thermal conductivity, further showcasing its superior insulation properties. Remarkably, the excellent thermal insulation performance of the CAFs is highlighted at an elevated temperature. As shown in Figure 4g, the CAF mats with multilayers have better thermal insulation properties. For the three-layer CAF mat, the |Δ*T*| is as high as 61 °C at a hot stage of 90 °C for 10 min, which is approximately 28, 36, and 40 °C higher than the corresponding values of the single-layered CAF, CT, and BC mats. Meanwhile, the detected temperature of the CAF mat was quite stable with a negligible increase even after 15 min, demonstrating the stable thermal insulating function of the CAFs (Figure 4h). Concurrently, to evaluate the heat-shielding performance of CAF-1 under ambient conditions, a CAF-1 mat was adhered to human skin for about 10 min. The infrared thermal image (Figure 4f) reveals that the surface temperature of CAF-1 closely matches the background temperature, indicating that the CAF-1 mat could potentially serve as a thermal stealth material. These observations suggest that CAFs exhibit enhanced thermal insulating capabilities compared with the other tested materials, which is crucial for potential applications in various fields.

### 2.5. α-Al_2_O_3_ Powder

**Figure 5:**
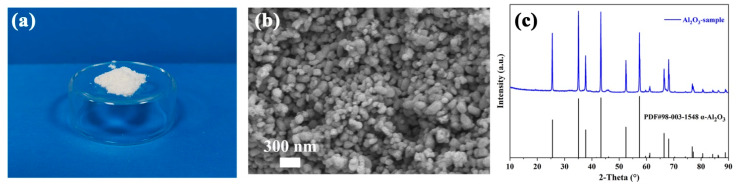
(**a**) Photograph and (**b**) SEM image of α-Al_2_O_3_ powder. (**c**) XRD patterns of α-Al_2_O_3_ samples.

Al_2_O_3_ was obtained through the calcination of Al(OH)_3_, and its chemical composition was analyzed. The Al_2_O_3_ content reached a remarkable level of 94.02 wt%, while the contents of Fe_2_O_3_, SiO_2_, and Na_2_O were measured to be 0.79, 2.59, and 0.78 wt%, respectively (Table S2). Consequently, the Al_2_O_3_ product exhibits exceptionally high purity. The prepared Al_2_O_3_ exhibits a macroscopic structure in the form of a white powder (Figure 5a). The microstructure of the α-Al_2_O_3_ was further thoroughly investigated using SEM, revealing a predominant morphology of spherical-like particles, with the majority of α-Al_2_O_3_ particles exhibiting a size distribution centered on approximately 100 nm (Figure 5b). Further calculation using the Williamson–Hall law via the High Score Plus software 4.9 (4.9.0.27512) reveals that the average grain size of α-Al_2_O_3_ is 126.4 nm. As shown in Figure 5c, the α-Al_2_O_3_ (corundum) phase is the predominant phase in the obtained Al_2_O_3_. This phenomenon may be attributed to the low activation energy of thermodynamically stable α-Al_2_O_3_, especially in the nanoscale form, which results in a lower sintering temperature and facilitating grain growth [55]. The above results indicated that by utilizing coal fly ash as a raw material, the silicon component therein can be transformed into aerogel material, while concurrently yielding high-purity α-Al_2_O_3_ products through aluminum extraction.

## 3. Experimental Section

### 3.1. Materials

Raw coal fly ash from the Baotou No.1 Thermal Power Plant (Inner Mongolia, China) was crushed and sieved to 100 mesh size (150 μm). Trimethylchlorosilane (TMCS, 98%), ethanol (99.7%), and n-hexane were obtained from Aladdin Reagent Co., Ltd. (Shanghai, China). Anhydrous sodium carbonate (Na_2_CO_3_) and sodium hydroxide (NaOH, ≥98%) were purchased from Beijing Chemical Reagent Co., Ltd. (Beijing, China). Concentrated sulfuric acid (H_2_SO_4_) was purchased from Demont Chemical Ltd. (Nanjing, China).

### 3.2. Preparation of the SiO_2_ Sol

The coal fly ash was mainly composed of SiO_2_ and Al_2_O_3_ in this experiment (Table S1), which existed as quartz and mullite, respectively (Figure S2). These compounds were chemically stable and required activation for further use. Coal fly ash was activated following a previously reported similar method [4,19,56]. Please see the Supporting Information for further details.

**Figure 6:**
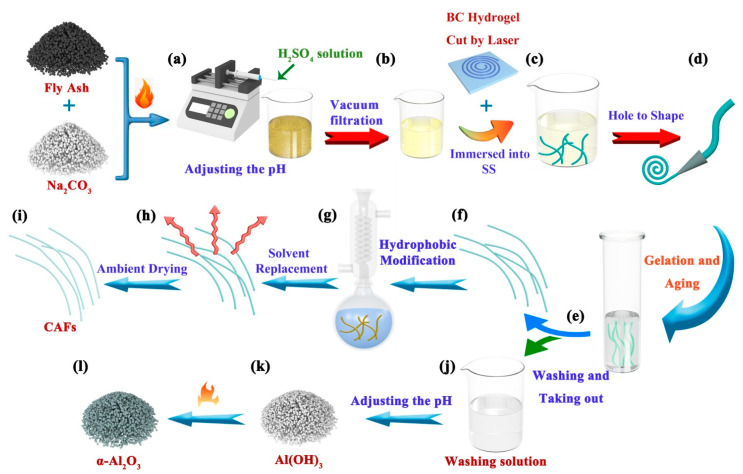
Schematic of the CAFs and α-Al_2_O_3_ preparation process. (**a**) The activated coal fly ash (AFA) was mixed with deionized water and the pH adjusted. (**b**) SiO_2_ sol (SS) was obtained via vacuum filtration. (**c**) The dried fiber-like BC matrixes are soaked in SS and (**d**) reshaped using a small hole mold. (**e**) The silica gel framework was developed within the BC matrix and then subjected to an aging process to reinforcing the gel skeleton, followed by washing with deionized water to remove metal ions. (**f**–**i**) The CAFs were obtained through hydrophobic modification, solvent replacement, and ambient drying of the wet gel fibers. (**j**–**l**) The pH of the washing solution was adjusted to precipitate Al(OH)_3_, which was subsequently subjected to calcination to yield α-Al_2_O_3_.

Activated coal fly ash (AFA, 5 g) was finely ground into powder. The powder was subsequently homogenized by adding 15 mL deionized water and adjusting the pH to 2 using a 6 mol/L H_2_SO_4_ solution, as illustrated in Figure 6a. The resulting mixture was magnetically stirred for 1 h to ensure the maximum dissolution of AFA in the acidic solution. Subsequently, SS was obtained via vacuum filtration, as depicted in Figure 6b. The residue obtained from the filtration process was dried, and the residue rate was calculated as the percentage of the drying residue mass to the initial mass of AFA (residue rate = drying residue mass/AFA mass × 100%). To achieve different concentrations, the obtained SS (~25 mL) was uniformly mixed with different volumes of deionized water, as indicated in Table 2.

### 3.3. Preparation of Bacterial Cellulose Matrix

Nata de coco slices, which were BC hydrogels with a thickness of 3.5 mm, were repetitively cleaned with deionized water to eliminate sugar residues. Subsequently, the cleansed nata de coco slices were subjected to a 4 h treatment at 90 °C in a sodium hydroxide (NaOH) solution, followed by thorough rinsing with deionized water until a neutral pH was achieved, resulting in the obtainment of purified BC hydrogel. The washed BC hydrogel was placed on a glass plate and mechanically compressed to remove excess water. A laser cutter with a power output of 15 W was employed to fabricate a fiber-like BC matrix with uniform dimensions of 2 mm width and approximately 500 mm length. Finally, the fiber-like BC matrix was obtained via freeze drying for 24 h after immersion in a mixed liquid solvent composed of water and tert-butanol (3:2 *v*/*v*).

### 3.4. Preparation of Silica–Bacterial Cellulose Composite Wet Gel Fibers

The dried fiber-like BC matrix was immersed in SS at different concentrations (Table 2 and Figure 6c). After sufficient diffusion for 2 h, the fiber-like BC matrix soaked with SS was passed through a tapered mold (Figure 6d). As a result, the fiber-like BC matrix containing the silica precursor became finer and more uniform. The molded fibers were placed in an airtight sample container with moist air (to create a humid atmosphere and prevent surface cracking of the samples) at 80 °C for 3.5 h. This step aimed to promote the conversion of silicate within the matrix into a silica gel skeleton and initiate an aging process, thereby strengthening the gel structure. Finally, the gelatinous fibers were washed in deionized water to remove Al^3+^, Fe^3+^, Na^+^, SO_4_^2+^, and excess H^+^ (Figure 6e) to obtain silica–BC composite wet gel fibers and a washing solution containing impurities (Figure 6f,j).

### 3.5. Hydrophobic Modification and Atmospheric Drying of Wet Gel Fibers to Obtain CAFs

The wet gel fibers were soaked in ethanol for 3 h, followed by substitution with n-hexane for an additional 3 h. These steps were performed to alleviate the rapid consumption of the hydrophobic modification agent within the gel pores. Subsequently, a flask was prepared by adding n-hexane (50 mL), triethylamine (TEA, 4 mL) (to neutralize HCl and prevent the hydrolysis of cellulose), and TMCS (3 mL). After solvent replacement, wet gel fibers (approximately 4 g in weight) were immersed in this solution. The flask was heated in an oil bath and refluxed for 2 h (Figure 6g). The wet gel fibers were subsequently immersed in an ethanol-filled beaker, which was refreshed every 30 min and repeated twice to eliminate the excess reagents and amine salts generated during the reaction. Subsequently, ethanol was replaced with n-hexane, and the aforementioned process was repeated (Figure 6h). Finally, the hydrophobically modified wet gel fibers were heated in an oven at 85 °C for 20 min to yield hydrophobic CAFs (Figure 6i).

### 3.6. Preparation of α-Al_2_O_3_ from the Extraction Raffinate of SiO_2_

First, 0.5 mol/L Na_2_CO_3_ was added dropwise into the washed solution containing impurities to adjust the pH = 6, followed by centrifuging (12,000 rpm, 10 min) to afford mixed sediments containing mainly Al(OH)_3_ and other metal hydroxide precipitates. Then, 0.1 mol/L NaOH was added dropwise to the mixed precipitates to adjust the pH = 12, and then centrifuged (12,000 rpm, 10 min) to separate the NaAlO_2_ solution and other precipitates. This step removed small quantities of Fe^3+^, Ca^2+^, Mg^2+^, etc., from the washed solution. Next, 0.5 mol/L sulfuric acid was dropped into the NaAlO_2_ solution until pH = 5.5 was reached, thereby yielding a sediment of Al(OH)_3_ (Figure 6k). Finally, Al(OH)_3_ was calcined in a muffle furnace at 1200 °C for 4 h to afford α-Al_2_O_3_ (Figure 6l).

### 3.7. Characterizations

X-ray fluorescence (XRF) and X-ray diffraction (XRD) analyses were performed to characterize the major elements and phases of coal fly ash, respectively. The morphologies and nanostructures of the samples were analyzed using scanning electron microscopy (SEM). The energy dispersive spectrum (EDS), contact angle, specific surface area, pore size distribution, mechanical properties, and chemical construction were determined. Finally, thermo-gravimetric analysis (TGA) and thermal insulation performance of the prepared materials were evaluated. Detailed characterization methods are provided in the Supplementary Materials.

## 4. Conclusions

Novel CAFs with high porosity, excellent mechanical properties, and superior thermal stability were prepared using coal fly ash as a raw material through a facile in situ sol-gel and ambient pressure drying process. Moreover, the extraction process also yields high-purity α-Al_2_O_3_ from the coal fly ash, facilitating the full utilization of this solid waste. The efficient formation of a silica gel skeleton within the BC matrix confers upon the CAFs’ exceptional thermal insulation properties and a significantly large specific surface area (588.75 m^2^/g). The CAFs possessed a high tensile strength (5.0 MPa), an ultimate elongation of 8.1%, and good weaving ability. Additionally, the stabilizing influence of silica on the BC matrix enables the CAFs to be utilized at relatively high temperatures, remaining thermally stable up to approximately 250 °C, and the typical IPN structure of CAFs bestows the fiber with a superior thermal insulation property under harsh environments. These characteristics demonstrate that the fabricated aerogel fiber is a potential candidate for next-generation high-performance multifunctional fibers. In conclusion, the presented method of preparing CAFs and α-Al_2_O_3_-based coal fly ash provide a new direction for the comprehensive use of coal fly ash.

## Figures and Tables

**Table 1:** Physical properties of CAFs.

Samples	SiO_2_ in Samples [% *w*/*w*]	Bulk Density [g/cm^3^]	S_BET_ [m^2^/g]	Pore Size [nm]	Porosity ^a^ [%]
CAF-1	57	0.1307	588.75	13.99	91.8
CAF-2	49	0.1238	564.31	12.64	90.1
CAF-3	35	0.1191	322.76	12.87	86.4
CAF-4	26	0.1092	234.81	12.41	81.5

^a^ The porosity encompasses voids resulting from crystal growth within gel skeletons during gel freezing.

**Table 2:** Volume of deionized water added to SS.

Solution	SS-1	SS-2	SS-3	SS-4
Added water (mL)	0	3	6	9
Corresponding sample name	CAF-1	CAF-2	CAF-3	CAF-4

## Data Availability

Data are contained within the article and Supplementary Materials.

## Supplementary Materials

The following supporting information can be downloaded at: https://www.mdpi.com/article/10.3390/molecules28247978/s1, References [57,58] are cited in the supplementary materials. Video S1: Video of a three-point bending test for CAF-3; Table S1: Elemental analysis of coal fly ash by XRF; Table S2: Elemental analysis of Al_2_O_3_ by XRF; Figure S1: The photos of the preparation process of CAFs: (a) BC hydrogel slice, (b) fiber-like BC hydrogel, (c) Drying fiber-like BC matrix, (d) the immersion of BC in SiO_2_ sol, (e) the secondary shaping of the BC matrix containing silica precursor, (f) the sample of CAF; Figure S2: XRD patterns of the raw coal fly ash; Figure S3: The reaction rate of coal fly ash under different parameters; Figure S4: XRD patterns of the AFA; Figure S5: The schematic chemical structure of bacterial cellulose; Characterization and Reference.
