# Supplementary material for: Recycling Coal Fly Ash for Super-Thermal-Insulating Aerogel Fiber Preparation with Simultaneous Al2O3 Extraction
Source: Molecules. 2023 Dec 6;28(24):7978. doi: 10.3390/molecules28247978 (PMC10746117; doi:10.3390/molecules28247978)
Supplement: Supplementary file 1 [file molecules-28-07978-s001.zip › molecules-2736044-supplementary.docx]

Recycling Coal Fly Ash for Super-Thermal-Insulating Aerogel Fiber Preparation with Simultaneous Al_2_O_3_ Extraction

Jie Gu ^1,2,3,†^, Lipeng Liu ^1,2,3,†^, Rongrong Zhu ^1,2^, Qiqi Song ^1,2,3^, Hanqing Yu ^1,2^, Pengjie Jiang ^1,2,3^, Changqing Miao ^1,2,3^, Yuxiang Du ^1,2,3^, Rui Fu ^1,2,3,^*, Yaxiong Wang ^1,2,3^, Yan Hao ^1,2,3^ and Huazheng Sai ^1,2,3,^*

^1^ School of Chemistry and Chemical Engineering, Inner Mongolia University of Science & Technology,
Baotou 014010, China; gujie22@mails.ucas.ac.cn (J.G.); lipengliu1998@163.com (L.L.);
rzr1208@163.com (R.Z.); songqiqiaa@163.com (Q.S.); q1252051550@163.com (H.Y.);
jpj1692787089@163.com (P.J.); qingmc@163.com (C.M.); duyuxiang5520@163.com (Y.D.);
wangyaxiong2021@126.com (Y.W.); haoyannk@163.com (Y.H.)

^2^ Inner Mongolia Key Laboratory of Coal Chemical Engineering & Comprehensive Utilization,
Inner Mongolia University of Science & Technology, Baotou 014010, China

^3^ Aerogel Functional Nanomaterials Laboratory, Inner Mongolia University of Science & Technology,
Baotou 014010, China

* Correspondence: furui14@mails.ucas.edu.cn (R.F.); shz15@tsinghua.org.cn (H.S.)

^†^ These authors contributed equally to this work.

**1. Characterization**

*1.1. XRF and XRD*

The major elements of the coal fly ash were analyzed using an X-ray fluorescence (XRF) spectrometer (Zetium, PANalytical, Holland). XRD analysis was performed using an X-ray diffractometer (Empyrean, PANalytical, Holland) at 40 kV and 40 mA using Cu Kα radiation; at a scan rate of 10°/min.

*1.2. Morphology and nanostructure*

The microstructure of the CAFs and Al_2_O_3_ samples were determined using scanning electron microscopy (SEM, Apreo 2, Thermo Scientific, America) aided by energy dispersive spectrum (EDS) analysis at an accelerating voltage of 10 kV and the working distance from 7.1 to 10.3 mm. The surfaces of the samples were prepared for analysis by simply sticking sample slices on the sample holder using a carbon pad, followed by coating with platinum.

*1.3. Surface wettability*

The surface wettability of the CAFs was measured using a contact angle measuring system (SZ-CAMC31, Xuanzhun, China). First, the CAFs were affixed to the sample holder using double-sided tape to ensure stability when the contact angles of the surfaces (internal and external) were measured. A droplet (2.5 μL) of deionized water was deposited on the surface of the CAFs. At least three measurements were obtained for each sample.

*1.4. Density Measurement*

Take the CAFs sample of 8 cm length, combined with the diameter given by SEM images to calculate the total volume of the sample (v_c_). Its mass (m_c_) was measured by a balance with a precision of 0.0001 g. The density of CAFs is calculated according to the ratio of mass to volume (𝑚_c_/v_c_).

*1.5. Nitrogen Physisorption Measurement*

The specific surface aera of the fibers were measured by the Brunauer‐Emmett‐Teller (BET) method [1] in the condition of the Nitrogen adsorption at pressures 0.01<p/p_0_<1.0. The Barrett‐Joyner‐Halenda (BJH) Analyses were conducted from desorption isotherm when the pore‐size distribution was investigated. Content of silica in the CAFs: The dried BC matrix of 5 cm in length was intercepted, its mass was weighed (𝑚_0_). Mass fraction (ω_s_) of silica could be calculated by the equation:

$$Mass fraction (\omega_{s})=\frac{m_{c}-m_{0}}{m_{c}}$$

*1.6. Porosity Measurement*

The porosity of CAFs was calculated according to Equation S2, where 𝜌_s_ and 𝜌_c_ are the bulk density of CAFs, the skeleton densities of pure silica aerogels and biopolymer (i.e. BC) matrix; ω_s_ and ω_c_ were the mass fraction of silica and BC in CAFs, respectively. Herein, based on literature data, the 𝜌_s_ and 𝜌_c_ were designed as 2.1 g cm^−3^ and 1.59 g cm^−3^ [2].

$$\mathrm{Porosity}\left( \% \right)=\left( 1-\frac{\omega_{s}}{{\omega_{s}\rho}_{c}-{\omega_{0}\rho}_{0}} \right)\times\%$$

*1.7. Thermal stability*

The thermal degradation curves were obtained using thermogravimetric analysis (TGA, STA449F3, NETZSCH, Germany). A sample weight of approximately 2 mg was obtained. The sample was placed in a ceramic pan and heated in the temperature range of 20-600°C at a rate of 10 °C/min under atmospheric pressure.

*1.8. Mechanical properties*

The mechanical properties of the fibers were tested by an electronic universal testing machine (HD‐B609B‐S, Guangdong, China), in the tensile mode. The strain rate was 2 mm/min for the tests. All the test samples were 2 cm. Each set of tensile strength test results are collected from at least five samples to obtain reliable values. The force was loaded at a speed of 2 mm/min for the three‐point bending tests with a fixture span of 15 mm.

*1.9. Thermal Insulation Measurement*

The thermographs were obtained by a thermal infrared camera (FLIR T620, Teledyne FLIR, Woburn, MA, USA). The camera was operated at a distance of about 30 cm. The CAFs sample were put on the heating plate, one thermos couple was connected to the sample and the other was on the hotplate next to the sample. Gradually heating up, the date acquisition device (34972A, Agilent, Santa Clara, CA, USA) was used to record the temperature of two thermos couples simultaneously.

**2. The Photos of the Preparation Process of CAFs**


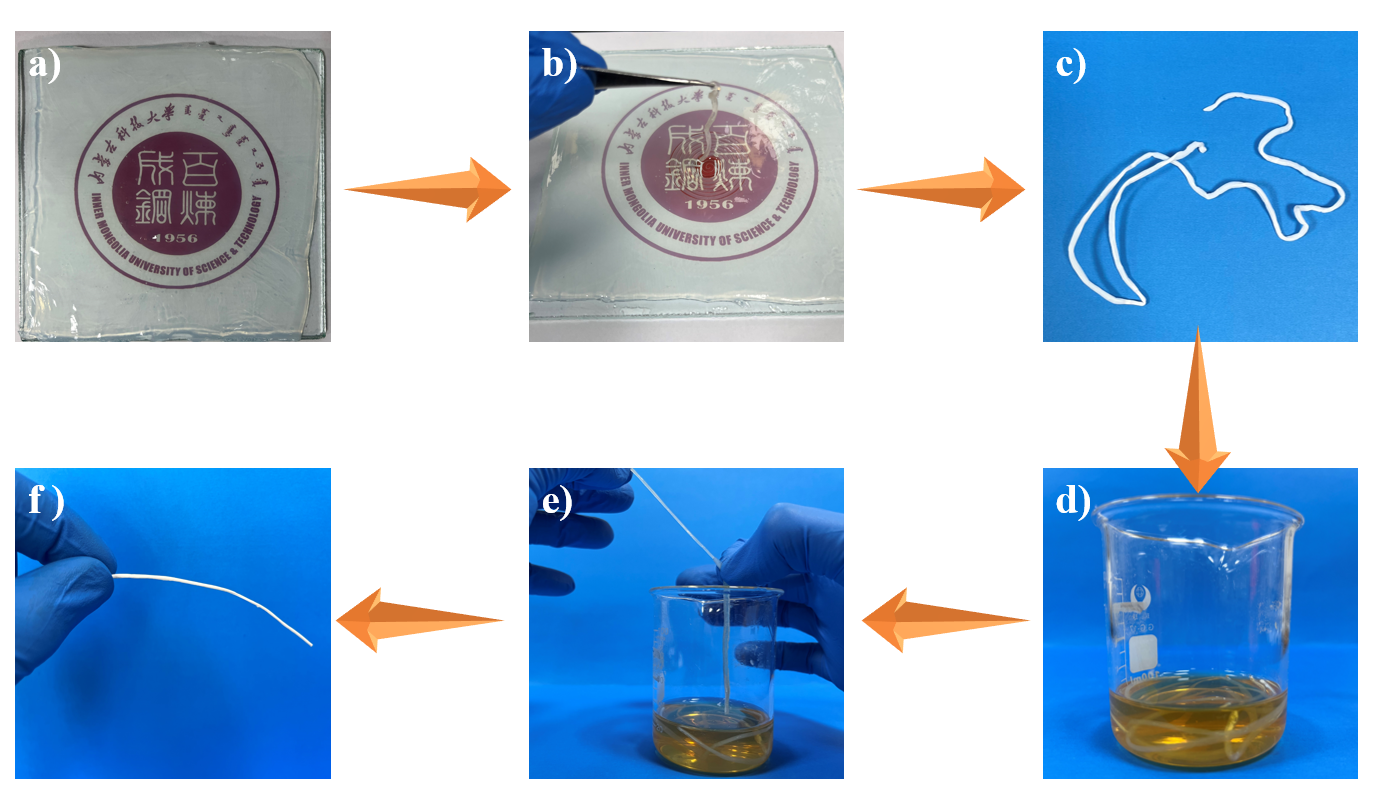


**Figure S1.** The photos of the preparation process of CAFs: (**a**) BC hydrogel slice, (**b**) fiber‐like BC hydrogel, (**c**) Drying fiber-like BC matrix, (**d**) the immersion of BC in SiO_2_ sol, (**e**) the secondary shaping of the BC matrix containing silica precursor, (**f**) the sample of CAF.

**3. The major elements and phase of the raw coal fly ash**

**Table S1.** Elemental analysis of coal fly ash by XRF.

| **Composition** | **SiO_2_** | **Al_2_O_3_** | **MgO** | **CaO** | **SO_3_** | **Fe_2_O_3_** | **TiO_2_** | **LOI^a^** |
| --- | --- | --- | --- | --- | --- | --- | --- | --- |
| (wt. %) | 47.61 | 41.02 | 0.40 | 1.84 | 1.19 | 4.59 | 1.65 | 1.7 |


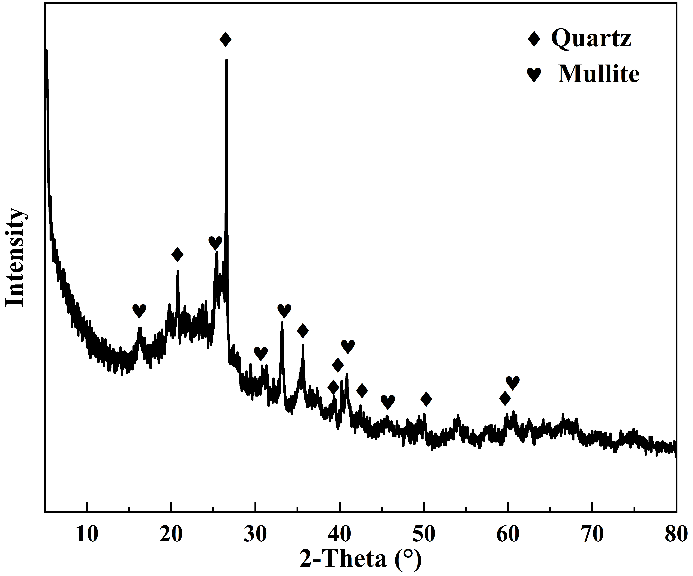


**Figure S2.** XRD patterns of the raw coal fly ash.

**4. Activation procedure of the coal fly ash**

The reaction rate of coal fly ash was calculated by the following equations:

$$Reaction Rate=\left[ 1-\frac{m'}{m\times(1-p)} \right]\times100\%$$

m is the original weight of coal fly ash and m' is the weight of the insoluble solid from the reaction production of the sinter (coal fly ash and Na_2_CO_3_) after mixing with 6 mol/L H_2_SO_4_ solution after adjusting pH=2 on a magnetic stirrer for 1 hour and p is the ignition loss of coal fly ash.

To obtain the high reaction rate of coal fly ash, three experimental parameters are studied, including weight ratio of coal fly ash to Na_2_CO_3_ (m (coal fly ash)/m (Na_2_CO_3_)), holding time, and calcination temperature. According to the literature, the coal fly ash samples and Na_2_CO_3_ were prepared by screening the ground raw materials to a mesh of 100 respectively. The weight ratio of coal fly ash to Na_2_CO_3_ was set as 13:8, 13:9, 13:10, 13:11, and 13:12 and then mixed evenly. Calcination times were set from 1.0 h to 3.5 h (each 30 min is a variable). The temperature was set to 700-950 °C (each 50^o^C is a variable). The values of three experimental parameters are determined by single variable method [3].


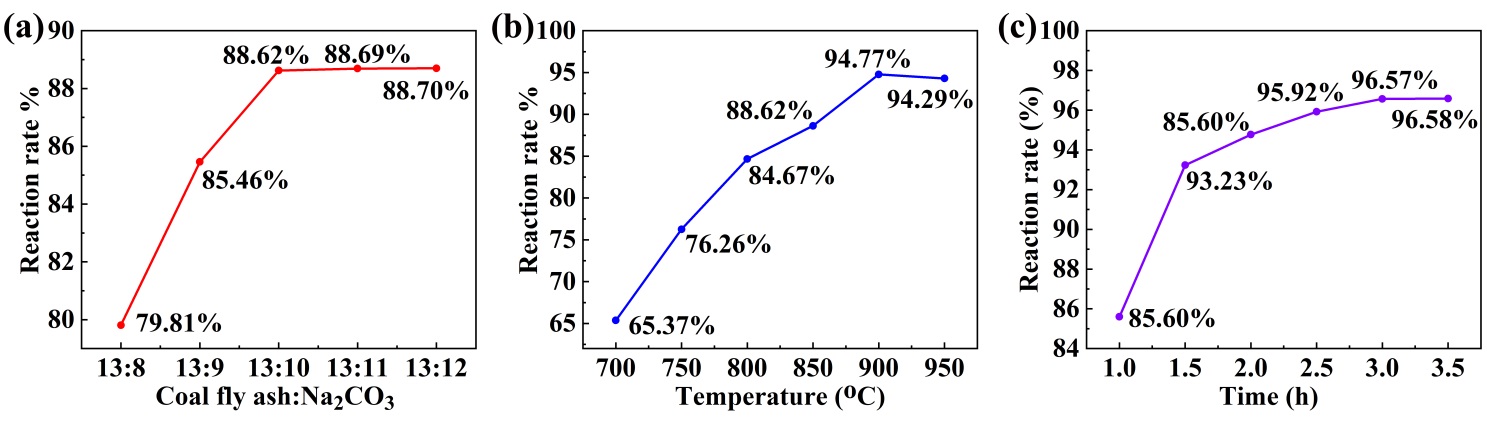


**Figure S3.** The reaction rate of coal fly ash under different parameters.

The raw coal fly ash was accurately weighed, and then mixed it with different masses of anhydrous Na_2_CO_3_ according to 13:8~13:12, subsequently calcined the mixture at 850°C for 2h. After the calcination reaction was completed, the activated coal fly ash (AFA) was cooled at room temperature, and then excess dilute sulfuric acid was added. The residue was washed with deionized water after vacuum filtration, and then dried. The calculated reaction rates were shown in Fig. S3 (a). By analyzing the data of AFA reaction rate, it could be concluded that when the mass ratio of coal fly ash and Na_2_CO_3_ was 13:10, AFA was adequately decomposed.

The optimum ratio of coal fly ash and Na_2_CO_3_ was 13:10 for mixing, the calcination time was 2h, and the calcination temperatures are 700°C, 750°C, 800°C, 850°C, 900°C, 950°C (The melting point of sodium carbonate is 851°C). As shown in Fig. S3 (b), it could be seen that the reaction rate was lower at 700°C -800°C. At this time, the Na_2_CO_3_ did not reach the melting point. Hence, the coal fly ash and Na_2_CO_3_ were solid-solid reaction. When the temperature reached 900°C, the reaction rate was importantly increased to 94.77%. At this time, the Na_2_CO_3_ was mostly molten state leading to a solid-liquid reaction, so the reaction rate was improved.

When the reaction temperature was set to 900 °C and the ratio of coal fly ash to Na_2_CO_3_ was 13:10, the reaction rate of coal fly ash at different times was explored. The reaction rate remained basically stable after 3.0 h (Fig. S3 (c)), indicating that the reaction was nearly complete and reached the maximum extent. The optimum calcination time was 3.0 h according to the analysis of the reaction rate.

In conclusion, the coal fly ash was with Na_2_CO_3_ at mass ratio of 13:10 and the activated coal fly ash was obtained calcined in 900^o^C for 3.0 h. The main component of AFA is NaAlSiO_4_ (Fig. S3) that can be decomposed in acid solution to obtain the SiO_2_ sol.


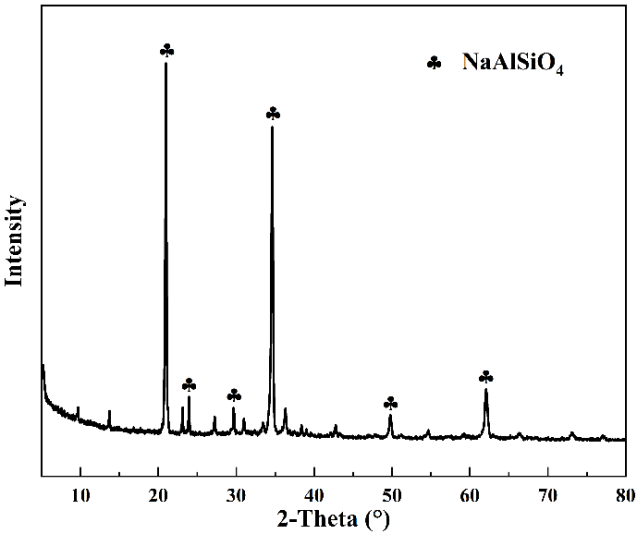


**Figure S4.** XRD patterns of the AFA.

**5. The major elements of the Al_2_O_3_**

**Table S2.** Elemental analysis of Al_2_O_3_ by XRF.

| **Composition** | **SiO_2_** | **Al_2_O_3_** | **Na_2_O** | **CaO** | **P_2_O_5_** | **Fe_2_O_3_** | **SO_3_** | **K_2_O** |
| --- | --- | --- | --- | --- | --- | --- | --- | --- |
| (wt. %) | 2.59 | 94.02 | 0.78 | 0.06 | 1.32 | 0.79 | 0.14 | 0.30 |

**6. The schematic chemical structure of bacterial cellulose**


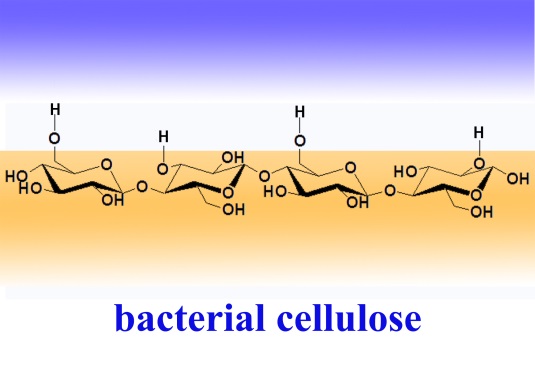


**Figure S5.** The schematic chemical structure of bacterial cellulose

**References**

1. Sai, H.; Fu, R.; Xing, L.; Xiang, J.; Li, Z.; Li, F.; Zhang, T. Surface Modification of Bacterial Cellulose Aerogels’ Web-like Skeleton for Oil/Water Separation. ACS Appl. Mater. Interfaces 2015, 7, 7373–7381, doi:10.1021/acsami.5b00846.

2. Heath, L.; Thielemans, W. Cellulose Nanowhisker Aerogels. Green Chem. 2010, 12, 1448, doi:10.1039/c0gc00035c.

3. Zhu, J.; Guo, S.; Li, X. Facile Preparation of a SiO_2_–Al_2_O_3_ Aerogel Using Coal Gangue as a Raw Material via an Ambient Pressure Drying Method and Its Application in Organic Solvent Adsorption. RSC Adv. 2015, 5, 103656–103661, doi:10.1039/C5RA20392A.
